# Gated Diffusion-controlled Reactions

**DOI:** 10.1186/2046-1682-4-4

**Published:** 2011-03-02

**Authors:** J Andrew McCammon

**Affiliations:** 1 Center for Theoretical Biological Physics, Department of Chemistry and Biochemistry, Howard Hughes Medical Institute, Department of Pharmacology, University of California at San Diego, La Jolla, CA 92093-0365, USA

## Abstract

The binding and active sites of proteins are often dynamically occluded by motion of the nearby polypeptide. A variety of theoretical and computational methods have been developed to predict rates of ligand binding and reactivity in such cases. Two general approaches exist, "protein centric" approaches that explicitly treat only the protein target, and more detailed dynamical simulation approaches in which target and ligand are both treated explicitly. This mini-review describes recent work in this area and some of the biological implications.

## 1. Gated Reactions

In science, it is recognized that solving one puzzle often reveals new puzzles. The determination of the first three-dimensional structure of a protein provided a famous example [1]. The protein involved was myoglobin, whose functions include the binding of oxygen molecules for storage and release as needed in muscle tissues. Inspection of a space-filling model of myoglobin immediately made clear that myoglobin could not function if the protein were rigid, since the polypeptide chain folded into a solid wall around the buried heme group to which the oxygen molecule binds; the same conundrum was soon found for hemoglobin. The puzzle of how ligands bind was discussed in a classic paper by Perutz and Mathews, in which they noted that there must be thermally excited fluctuations in the protein structures that enable ligand molecules to migrate between the external solvent and the heme binding groups [2].

Subsequent studies of protein structure and function have shown that structural fluctuations are, in fact, often required for ligand binding and release [3]. An early analysis of the kinetic consequences of such "gated" binding processes was presented by McCammon and Northrup in 1981 [4]. This revealed two limiting cases, for a binding site that is highly reactive when exposed. In the fast gating limit, for which opening and closing of the gate is much faster than the rate of escape of ligand from the neighborhood of the gate into the surrounding solvent, the rate of binding approaches that of the always-open protein. In the slow gating limit, for which opening and closing of the gate is much slower than the rate of escape of the ligand, the rate approaches that of the always-open protein multiplied by the fraction of the time the gate is open.

The years since 1981 have seen marked improvements in the theoretical underpinnings for the analyses of gated diffusion-controlled reactions, and applications of these theories to a wide range of biomolecular systems. Much of this work, like that of McCammon and Northrup [4], focuses on the protein: Simulations or experimental data on the gate dynamics yield an opening/closing timescale that is compared with the timescale for ligand escape from the neighborhood of the gate. A more detailed but more demanding approach involves explicit treatment of ligand and protein in a combined model, as in an early study by Wade et al. [5]. This mini-review briefly describes the state of such research, beginning with the protein-centric models.

## 2. Theoretical Advances in the Protein-centric Models

Szabo et al. provided a model for gated diffusion-controlled reactions by including a stochastically gated sink term in the familiar diffusion equation [6]. For a simple spherical model of a protein with isotropic reactivity at its surface, the equation is centrosymmetric,

(1) $$\frac{\partial \rho }{\partial t}=r^{-2}\frac{\partial }{\partial r}\left(r^{2}D\frac{\partial \rho }{\partial r}\right)-k_{s}h(t)\rho \delta (r-R)$$

Here, *ρ*(r,t) is the density of ligands at a distance *r *from the center of the protein at time *t*, *D *is the relative diffusion constant of ligand and receptor, *k_s _*is the specific rate constant in the gate-open (reactive) state, *R *is the ligand-protein contact distance, and *h*(*t*) is a characteristic gating function that fluctuates between values of 0 (gate closed) and 1 (gate open). Equation 1 is supplemented with a reflecting wall boundary condition at *r *= *R*. If the reaction is fully diffusion-controlled when the gate is open, *k_s _*= ∞, corresponding to a fully absorbing boundary condition. Given the solution of Eq (1), the average binding rate that would be observed experimentally is characterized by the bimolecular rate constant,

(2) $$k=\langle 4\pi R^{2}k_{s}h(t)\rho (R,t)\rangle$$

where the brackets indicate a time average. For the fast gating case as defined above and *k_s _*= ∞, one recovers the familiar Smoluchowski expression for a perfectly absorbing sphere, *k *= 4*πDR*. For the corresponding slow gating case, the rate constant is just the Smoluchowski result multiplied by the fraction of the time the gate is open. In addition to the rather idealized case of a fully reactive spherical surface, Szabo et al. also derived results for a reactive circular patch on a planar surface (approximating a reactive site on a membrane) and a similar patch on an otherwise nonreactive spherical surface (approximating an active site in an enzyme) [6].

Zhou subsequently derived rate equations for a more general and realistic set of models, in which the reactive surface is represented as a patch that is recessed below the major surface of the protein and only accessible by a channel from that surface [7]. As in Szabo et al. [6], binding is stochastically gated according to a simple Markovian scheme,

(3) $$\text{Open}\overset{w_{c}}{\underset{w_{0}}{⇄}}\text{Closed}$$

where *w*_*c *_and *w*_*o *_correspond to the rates of gate closing and opening, respectively. Zhou's derivation yields

(4) $$\frac{1}{k}=\frac{1}{k_{\text{Open}}}+\frac{w_{c}}{w_{o}w\hat{k}(w)}$$

where *w *= *w_c _*+ *w_o_*. In Eq (4), *k *is the rate constant for ligand binding in the presence of gating, *k_Open _*is the corresponding rate constant if the gate has been fixed open for a long enough period to achieve steady state conditions, and $\hat{k}(s)$ is the Laplace transform of the total flux across the entrance to the active site in the absence of a gate if the initial distribution of ligands is an equilibrium distribution outside the entrance (i.e., the distribution that would develop if the gate were closed for a long time). The limiting rate constants are similar to what has been described before, but now we express these symbolically. Fast or slow gating is judged relative to the time required for ligands to diffuse away from the entrance to the site of gating and reaction; the latter time can be approximated as

(5) $$\tau_{D}\approx R^{2}/D$$

Thus, in the limit of fast gating, defined by *w*^-1 ^≪ *τ_D_*, one has *k *≈ *k*_Open_. In the limit of slow gating, defined by *w*^-1 ^≫ *τ_D_*, one has *k *≈ (*w_o_*/*w*)*k*_Open_.

## 3. Applications of the Protein-centric Models

### 3.1. Acetylcholinesterase

As mentioned at the outset, myoglobins and hemoglobins seem unable to perform their functions of binding oxygen, based on their crystal structures alone. A similar situation became apparent with the first crystallographic studies of acetylcholinesterases [8,9]. These enzymes are responsible for inactivating the neurotransmitter acetylcholine in synapses and neuromuscular junctions, so that they have been under tremendous evolutionary pressure to operate with great speed, as indeed they are known to do [10-12]. It was therefore a surprise to see in the crystal structures that the active site is deeply buried at the bottom of a 2 nm deep gorge in the protein, and that there is a bottleneck in the gorge that would not allow passage of the acetylcholine molecule if the protein were rigid. Molecular dynamics studies helped to resolve this paradox by showing that thermal fluctuations open and close the bottleneck, and - indeed - suggest the existence of transient alternate passageways [13-17]. Careful analysis of one of these simulations indicated that the timescale for gate opening/closing (gauged by fluctuations in the width of the bottleneck in the gorge, relative to the width of acetylcholine) is roughly 0.5 ps, while the timescale for diffusion of acetylcholine away from the enzyme is roughly 10 ns [18]. Thus, acetylcholinesterase is solidly within the fast gating regime, consistent with the great speed of the enzyme. It was also suggested that gating could provide a dynamic mechanism for selectivity, since potential substrates that are only modestly larger than acetylcholine rarely encounter an opened bottleneck, and their rates of catalysis are reduced by orders of magnitude [18]. The special role of aromatic sidechains in the bottleneck dynamics - enabling substantial width fluctuations with local torsion angle fluctuations - has also been discussed [16] and noted in other gating channels and enzymes [3]. Because larger scale, collective motion within the protein also contributes to the gate dynamics, it has been noted that these motions could represent a new mechanism for allosteric phenomena. The binding of a ligand in one part of a protein could alter the gating motions and binding selectivity elsewhere [16].

So far, the discussion in this section has concerned only monomeric forms of acetylcholinesterase. In the setting of synapses and neuromuscular junctions, the enzyme often occurs as homotetramers, with some of the active sites potentially occluded by steric interactions with adjacent monomers. Thus, gating occurs at the level of quaternary structure as well as tertiary structure. Simulations suggest that the quaternary dynamics is actually in the slow gating regime, with opening/closing on the 50 ns timescale, again compared with ligand escape on the 10 ns timescale. However, the active sites seem only to be occluded about 15% of the time, so slow gating does not involve a substantial penalty in kinetic terms - the rate is reduced only by about 15% from the always-open ideal case [19,20].

### 3.2. Messenger RNA Capping Enzymes

Messenger RNA molecules are normally capped at the 5' end soon after their transcription has begun. The capping groups are needed to protect the nascent mRNA from degradation by RNases, and also to enable recognition of the mRNA by the ribosome. Studies of the dynamics of a virally encoded mRNA capping enzyme have been reported by Swift and McCammon [21]. The enzyme comprises two major globular domains linked by a hinge region. GTP nucleotides must have access to the cleft between the two large domains for the capping reaction to proceed. The simulation studies on the enzyme show that it samples a closed state and a somewhat open state that are also seen in crystal structures. But the simulations also suggest the existence of a hyper-open state, which is the one that allows facile binding of GTP. Analyses of the dynamics of the system with simple estimates of the diffusional dynamics of the enzyme's hinge-bending motion suggest that the enzyme operates in the fast gating regime. The gate opening/closing timescale is estimated to be about 3.3 ns, and the timescale for GTP to escape is estimated to be about 130 ns. Thus, even though the enzyme is found to prefer relatively closed structures, it seems to sample the hyper-open state frequently enough to allow near optimal reactivity.

## 4. Explicit Treatment of Protein and Ligand

Treating the ligand explicitly in addition to the protein clearly is more demanding of computational resources, but offers the advantage of greater realism. In particular, the interactions between these species can be treated in some detail. This may be important for the separated species, e.g., to allow for higher-order multipole components of electrostatic coupling, possibly varying with conformational fluctuations of the ligand. But, it is likely to be particularly important in the encounter complex, where steric interactions between the flexible species may strongly perturb the gating dynamics and the entry of the ligand to the reactive site. These might be colorfully described as "foot in door" effects. This level of treatment has been attempted in a few Brownian dynamics simulations, but is likely to become more common.

### 4.1. Triosephosphate Isomerase

Wade et al. conducted Brownian dynamics simulations of a model substrate interacting with a flexible, gated enzyme that is known to exhibit diffusion-controlled kinetics [5]. The enzyme is chicken muscle triosephosphate isomerase, a homodimeric structure with flexible polypeptide loops gating the two active sites. In the computer model, the enzyme subunits were taken to be rigid except for the flexible loops, which were represented as chains of 17 spherical residues, of which the central 11 residues were allowed to move. The substrate, glyceraldehyde 3-phosphate (GAP) was represented as a pair of touching spheres of radius 0.2 nm with central charges of -0.3 e and -1.7 e to represent the glyceraldehyde and phosphate moieties, respectively. The charges account for the monopole and dipole moments of the doubly-charged form of GAP, and hydrodynamic interactions between the two spheres were included to give the correct diffusion constant for the substrate. Electrostatic and steric interactions between GAP and the rigid and flexible portions of the enzyme were included by use of a coarse-grained model, and an ionic strength of 0.1 M was assumed.

The Brownian dynamics simulations were performed with the Ermak-McCammon algorithm [22]. Analysis of the trajectories showed that both translational and rotational electrostatic steering contributed significantly to the kinetics of successful docking of the substrate to the active site. More importantly from the perspective of gating, the opening and closing of the flexible loops of the unliganded enzyme is seen to be on a timescale of about 1 ns, and the diffusional relaxation time of the substrate is on a timescale of about 16 ns. Thus, the system is in the fast gating regime, and the estimated rate constant is close to that observed experimentally. The conclusions therefore support those of an earlier "protein centric" study [23], but the explicit simulation shows clearly how a substrate may approach a closed active site and remain nearby long enough for the site to open so that reaction can ensue. It should be noted that some more recent studies (reviewed in [24]) suggest slower gating motions and that simulations of other peptide loop motions are somewhat slower (see below). It is possible that approximations in this pioneering study yielded somewhat rapid loop motions, though additional study is clearly warranted. In any case, since all studies point to the gate being open at least half of the time in the unliganded system, there would still only be a small departure from the always-open rate, however, even in the limit of slow gating.

### 4.2. HIV-1 Protease

HIV-1 Protease, an essential enzyme for HIV replication and an important target of antiviral drugs, is a small homodimeric protein with two polypeptide "flaps" covering the active site at the dimer interface. Chang et al. used coarse-grained models of the enzyme and a nonapeptide substrate to study the gating of substrate binding by the opening and closing of the flaps [25]. As in the study of triosephosphate isomerase by Wade et al. described above [5], Brownian dynamics was used to simulate the internal and overall diffusion the flexible molecules [22]. Again, the coarse-grained model comprised spherical interaction centers for the amino acid residues, but the parameters for this model were developed by reference to a large number of experimental structures of the protease [26,27]. With these methods, it was possible to run many encounter trajectories on the microsecond timescale.

Analysis of the results showed that the gating timescale was of the order of 60 ns, while the timescale for diffusional escape of the peptide substrate was of the order of 40 ns [25]. Thus, the rate of binding is somewhat reduced by the gating. Interestingly, the fractions of gate-open time were found to be about 14% and 2% for the wild-type enzyme and a drug resistant mutant (G48V/V82A/I84V/L90M), raising the possibility that slowed binding of large antiviral drugs could contribute to resistance in the mutant [25].

It is worth noting that "protein centric" studies of gating in HIV-1 protease have also been reported. These have shown that the experimental order of binding rate constants for different antiviral drugs can be rationalized in the slow-gating picture [28] and that the effects of crowding by other macromolecules alter the gating dynamics and, presumably, the kinetics of ligand binding [29,30].
